# Arcuate AgRP, but not POMC neurons, modulate paraventricular CRF synthesis and release in response to fasting

**DOI:** 10.1186/s13578-022-00853-z

**Published:** 2022-07-28

**Authors:** Alan Carlos Alves Fernandes, Franciane Pereira de Oliveira, Gimena Fernandez, Luane da Guia Vieira, Cristiane Gugelmin Rosa, Taís do Nascimento, Suzelei de Castro França, Jose Donato, Kristen R. Vella, Jose Antunes-Rodrigues, André Mecawi, Mario Perello, Lucila Leico Kagohara Elias, Rodrigo Rorato

**Affiliations:** 1 Department of Biotechnology, University of Ribeirao Preto, Ribeirão Prêto, SP 14096-900 Brazil; 2 Department of Physiology, Ribeirao Preto Medical School, University of Sao Paulo, Ribeirão Prêto, SP 14049-900 Brazil; 3 Department of Physiology and Biophysics, Institute of Biomedical Sciences, University of Sao Paulo, São Paulo, SP 05508-000 Brazil; 4 Department of Endocrinology, Diabetes and Metabolism and the Weill Center for Metabolic Health, Weill Cornell Medical College, New York, NY 10021 USA; 5 Laboratory of Neurophysiology of the Multidisciplinary Institute of Cell Biology [IMBICE, Argentine Research Council (CONICET) and Scientific Research Commission, Province of Buenos Aires (CIC-PBA), National University of La Plata, La Plata, 403, Buenos Aires, Argentina; 6 Department of Biophysics, Paulista Medical School, Federal University of Sao Paulo, São Paulo, SP CEP 04023-062 Brazil

**Keywords:** HPA axis, CRF, Fasting, AgRP, DREADD

## Abstract

**Background:**

The activation of the hypothalamic–pituitary–adrenal (HPA) axis is essential for metabolic adaptation in response to fasting. However, the neurocircuitry connecting changes in the peripheral energy stores to the activity of hypothalamic paraventricular corticotrophin-releasing factor (CRF^PVN^) neurons, the master controller of the HPA axis activity, is not completely understood. Our main goal was to determine if hypothalamic arcuate nucleus (ARC) POMC and AgRP neurons can communicate fasting-induced changes in peripheral energy stores, associated to a fall in plasma leptin levels, to CRF^PVN^ neurons to modulate the HPA axis activity in mice.

**Results:**

We observed increased plasma corticosterone levels associate with increased CRF^PVN^ mRNA expression and increased CRF^PVN^ neuronal activity in 36 h fasted mice. These responses were associated with a fall in plasma leptin levels and changes in the mRNA expression of *Agrp* and *Pomc* in the ARC. Fasting-induced decrease in plasma leptin partially modulated these responses through a change in the activity of ARC neurons. The chemogenetic activation of POMC^ARC^ by DREADDs did not affect fasting-induced activation of the HPA axis. DREADDs inhibition of AgRP^ARC^ neurons reduced the content of CRF^PVN^ and increased its accumulation in the median eminence but had no effect on corticosterone secretion induced by fasting.

**Conclusion:**

Our data indicate that AgRP^ARC^ neurons are part of the neurocircuitry involved in the coupling of PVN^CRF^ activity to changes in peripheral energy stores induced by prolonged fasting.

**Supplementary Information:**

The online version contains supplementary material available at 10.1186/s13578-022-00853-z.

## Background

The hypothalamic–pituitary–adrenal (HPA) axis consists of hypophysiotropic corticotropin-releasing factor (CRF) neurons, localized in the medial parvocellular subdivision of the PVN (CRF^PVN^), that control the synthesis and secretion of ACTH from the anterior pituitary that in turn modulates the glucocorticoid secretion (e.g., corticosterone in rodents) from the adrenal gland [1]. As the major HPA axis regulator, CRF^PVN^ neurons are responsible for orchestrating endocrine, autonomic and behavioral responses to stress [2–4].

Increased HPA axis activity is observed in response to different kinds of clinical metabolic disorders, such as obesity, metabolic syndrome, type 1 and 2 diabetes, and HIV-related lipodystrophy [5–7]. Despite the differences in the pathogenesis of these conditions, it has been suggested that changes in peripheral energy stores or the inability of the organism to sense them, underlie the changes in the HPA axis activity [8]. Reinforcing the coupling of the HPA axis with changes in peripheral energy stores, it was demonstrated that leptin administration reduces corticosterone in hypoleptinemic type 1 diabetic animals [9–11] and restores fasting-increased plasma corticosterone levels in mice [9].

The central nervous system (CNS) is considered the critical site for leptin’s actions on food consumption and energy expenditure [12]. Leptin receptor (LepR) is not expressed in CRF^PVN^ neurons [13] but it is highly expressed in brain sites sensitive to changes in peripheral energy stores, such as the hypothalamic arcuate nucleus (ARC), where LepR colocalizes with a subset of proopiomelanocortin (POMC) or agouti-related protein (AgRP) neurons (POMC^ARC^ or Agrp^ARC^ neurons, respectively) [14]. In the ARC, POMC is predominantly cleavaged to alpha-melanocyte stimulating hormone (α-MSH) that negatively modulates the energy homeostasis [10]. On the other hand, the activation of AgRP^ARC^ neurons, which also produce neuropeptide Y (NPY) and GABA, increases food intake and reduces energy expenditure [10]. Additionally, pharmacological studies have demonstrated that α-MSH, AgRP and NPY signaling can modulate the HPA axis activity [15, 16]. The ARC neurons are both directly and indirectly connected to the PVN [17, 18]. However, in addition to the ARC, these neuropeptides/neurotransmitters are also expressed in other brain nuclei [19–21].

Therefore, in the present study we aimed to determine if POMC^ARC^ and/or Agrp^ARC^ neurons can communicate fasting-induced changes in peripheral energy stores to CRF^PVN^ neurons.

## Results

### Fasting activates the HPA axis

Initially, the effects of 24 h and 36 h fasting were compared. Both fasting periods reduced the body weight, plasma leptin and blood glucose levels, and increased plasma corticorterone concentration; however, only 36 h fasting increased mRNA expression of *Crf* in the PVN and *Agrp* and *Npy* in the ARC, as compared to 24 h fasting (Additional file 2: Fig. S2). Therefore, the additional studies were performed in 36 h-fasted animals.

**Fig. 1:**
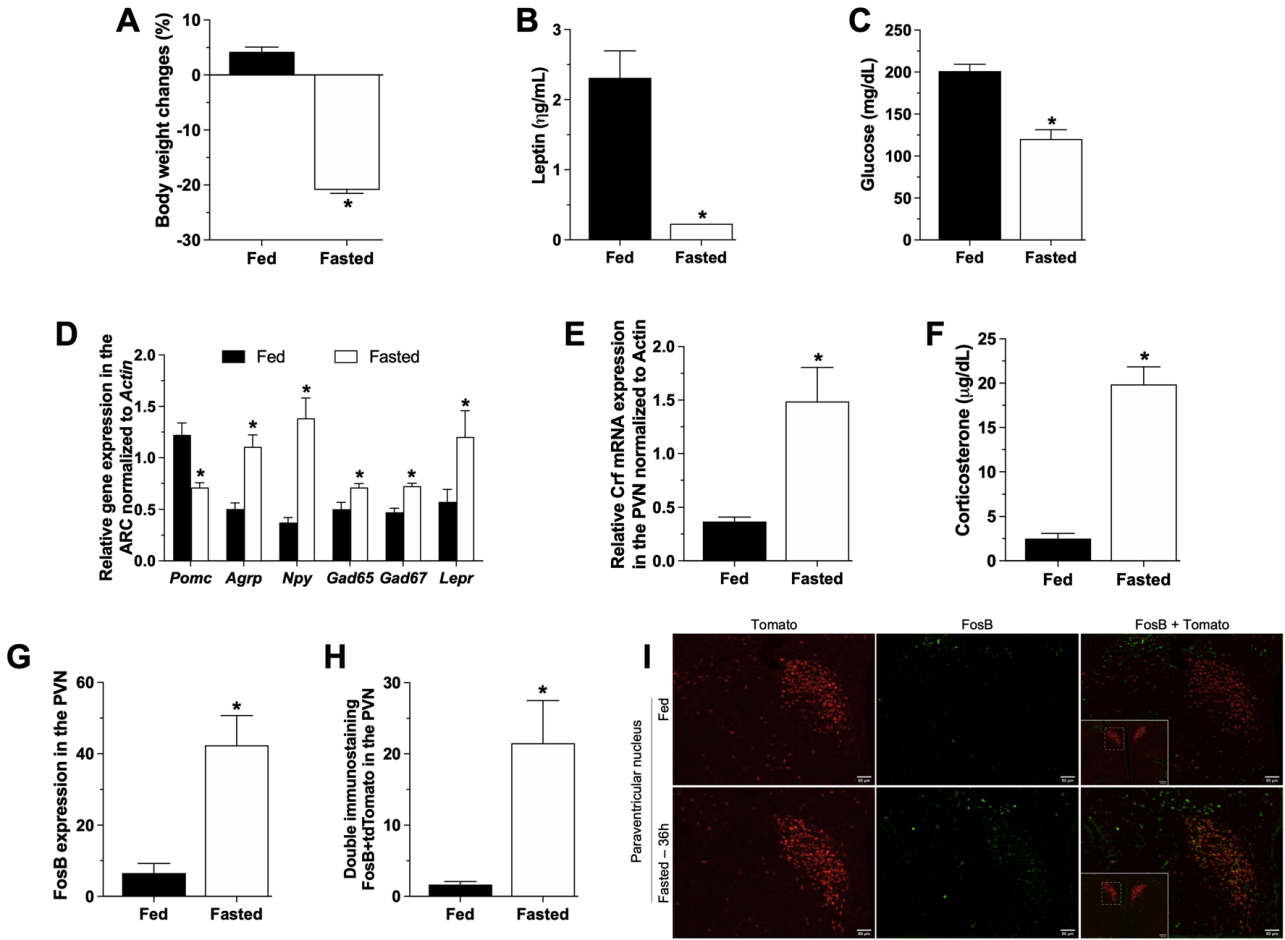
Fasting activates the HPA axis: Body weight change (**A**), plasma leptin (**B**) and glucose (**C**) levels, ARC gene expression (**D**), *Crf* gene expression in the PVN (**E**), plasma corticosterone levels (**F**), FosB (**G**) and double FosB/tdTomato (**H**) immunostaining in the PVN in fed and 36 h-fasted adult (8–10 weeks) male mice (n = 8–10) euthanized at 10am as described in the Additional file 1: Fig. S1. Data from graphs A to F were obtained in WT animals, while graphs G and H and the photomicrographs (×20, insert ×10) in **I** were obtained from *Crh-IRES-Cre*::tdTomato (red) mouse and submitted to immunostaining for FosB (green). Data are expressed as mean ± SEM and were analyzed by unpaired t-test or Mann-Whitey. *p < 0.05 when comparing fasting vs fed mice

We observed body weight loss (Fig. 1A, fed = 4.2 ± 0.8%BWC and fasted = − 20.9 ± 0.6%BWC, Mann–Whitney test, *p* = 0.001) associated with increased plasma corticosterone levels (Fig. 1F, fed = 2.5 ± 0.5 µg/dL and fasted = 19.8 ± 1.9 µg/dL, unpaired *t* test, *p* < 0.0001), mRNA expression of *Crf* in the PVN (Fig. 1E, fed = 0.3 ± 0.0 AU and fasted = 1.4 ± 0.3 AU, unpaired *t* test, *p* = 0.01) and increased CRF^PVN^ neuronal activity (Fig. 1H, fed = 1.7 ± 0.4 FosB + tdTomato and fasted = 21.5 ± 6 FosB + tdTomato, unpaired *t* test, *p* = 0.001) in 36 h fasted mice compared to fed mice. As expected, we also observed reduced plasma glucose (Fig. 1C, fed = 201 ± 8.1 mg/dL and fasted = 120 ± 11.1 mg/dL, unpaired *t* test, *p* < 0.0001) and leptin (Fig. 1B, fed = 2.3 ± 0.3 ng/mL and fasted = 0.2 ± 0.0 ng/mL, unpaired *t* test, *p* = 0.002) levels in 36 h fasted mice. Additionally, in the ARC of 36 h-fasted animals, we observed increased mRNA expression of *Agrp* (fed = 0.5 ± 0.3 AU and fasted = 1.1 ± 0.1 AU, unpaired *t* test, *p* = 0.003)*, Npy* (fed = 0.3 ± 0.0 AU and fasted = 1.3 ± 0.1 AU, unpaired *t* test, *p* = 0.003)*, Gad65* (fed = 0.5 ± 0.0 AU and fasted = 0.7 ± 0.0 AU, unpaired *t* test, *p* = 0.02), *Gad67* (fed = 0.4 ± 0.0 AU and fasted = 0.7 ± 0.0 AU, unpaired *t* test, *p* = 0.0008) and Lepr (fed = 0.6 ± 0.1 AU and fasted = 1.2 ± 0.2 AU, Mann–Whitney test, *p* = 0.03) , and decreased *Pomc* mRNA expression (fed = 1.2 ± 0.1 AU and fasted = 0.7 ± 0.0 AU, unpaired *t* test, *p* = 0.003) (Fig. 1D).

### Leptin treatment prevents HPA axis activation under fasting

**Fig. 2:**
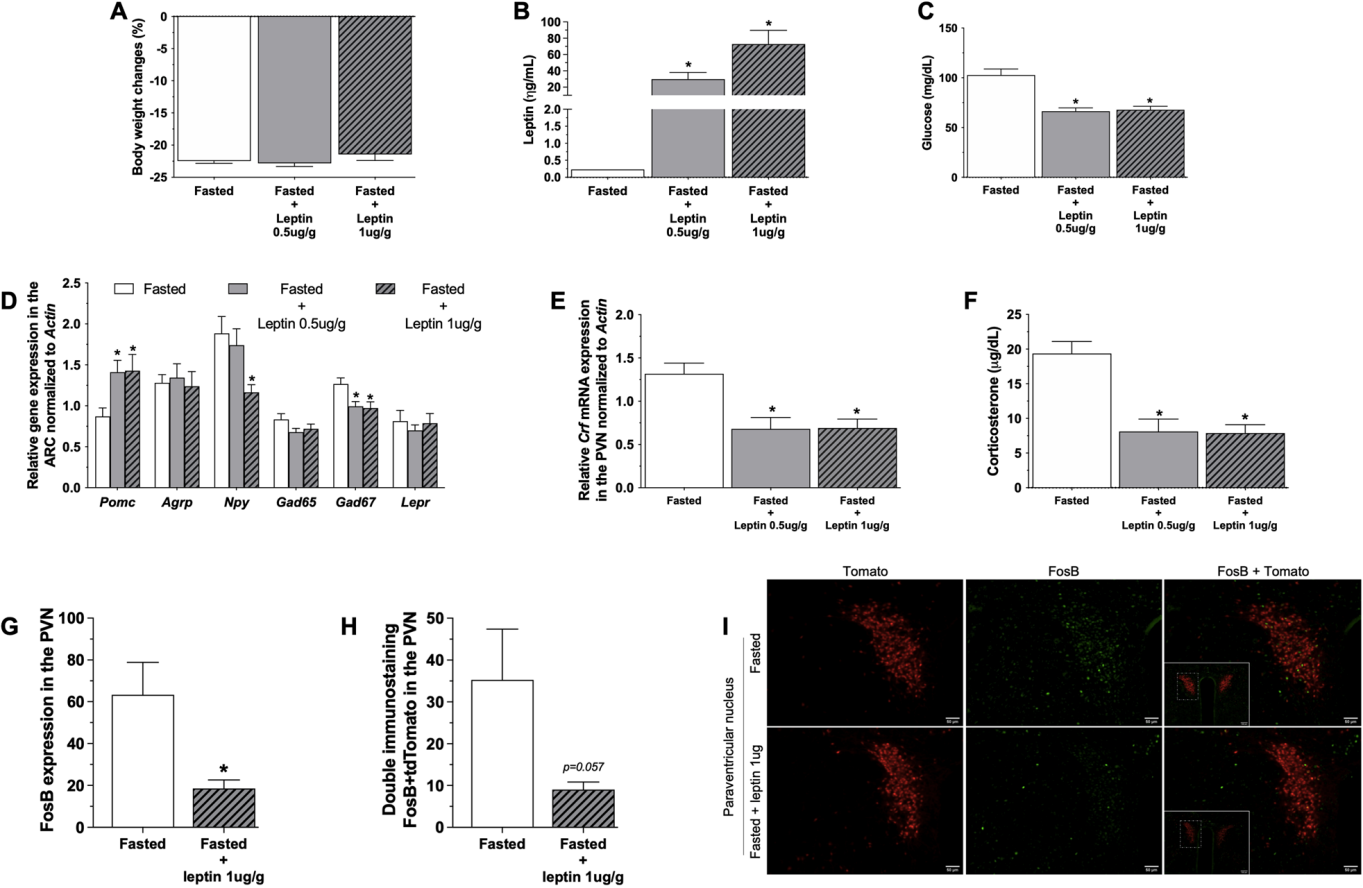
Leptin treatment prevents HPA axis activation under fasting: Body weight change (**A**), plasma leptin (**B**) and glucose (**C**) levels, ARC gene expression (**D**), *Crf* gene expression in the PVN (**E**), plasma corticosterone levels (**F**), FosB (**G**) and double FosB/tdTomato (**H**) immunostaining in the PVN in 36 h-fasted adult (8–10 weeks) male mice (n = 10–12) treated with 4 injections (each 8 h) of saline, 0.5 µg/g or 1 µg/g of leptin (i.p.) and euthanized at 10 a.m. as also described in the Additional file 1: Fig. S1. Data from graphs **A** to **F** were obtained in WT animals, while graphs **G** and **H** and the photomicrographs (×20, insert ×10) in **I** were obtained from *Crh-IRES-Cre*:tdTomato (red) mouse and submitted to immunostaining for FosB (green). Data are expressed as mean ± SEM and were analyzed by one-way ANOVA followed by post hoc Tukey’s multiple comparison or Kruskall–Wallis test followed by Dunn’s multiple comparison. *p < 0.05 when comparing leptin 0.5 µg/g or 1 µg/g vs saline treatment in fasted mice

Leptin treatment robustly increased plasma leptin levels (Fig. 2B, fasted + saline = 0.23 ± 0.0 ng/mL, fasted + 0.5 leptin = 29.9 ± 8.2 ng/mL and fasted + 1.0 leptin = 72.7 ± 17 ng/mL, Kruskall–Wallis test, *p* < 0.05) but had no effect on fasting-induced body weight loss (Fig. 2A, fasted + saline = − 22.5 ± 0.3%BWC, fasted + 0.5 leptin = − 22.8 ± 0.5%BWC and fasted + 1.0 leptin = − 21.5 ± 0.9%BWC, Kruskall–Wallis test, *p* = 0.003). Additionaly, leptin treatment reduced fasting-induced corticosterone secretion (Fig. 2F, fasted + saline = 19.4 ± 1.7 µg/dL, fasted + 0.5 leptin = 8.1 ± 1.8 µg/dL and fasted + 1.0 leptin = 7.8 ± 1.2 µg/dL, one-way ANOVA followed by post hoc Tukey’s multiple comparison, *p* < 0.001), associated with reduction of *Crf* mRNA expression (Fig. 2E, fasted + saline = 1.3 ± 0.1 AU, fasted + 0.5 leptin = 0.6 ± 0.1 AU and fasted + 1.0 leptin = 0.7 ± 0.1 AU, one-way ANOVA followed by post hoc Tukey’s multiple comparison, *p* = 0.002). Leptin treatment (1 µg/g) also reduced the fasting-induced FosB expression in CRF^PVN^ neurons (Fig. 2H*,* fasted + saline = 35.2 ± 12 FosB + tdTomato and fasted + leptin 1 µg/g = 9 ± 1.8 FosB + tdTomato, unpaired *t* test, *p* = 0.057). We also observed reduced plasma glucose levels (fasted + saline = 102 ± 6.1 mg/dL, fasted + 0.5 leptin = 66.3 ± 3.3 mg/dL and fasted + 1.0 leptin = 67.8 ± 3.4 mg/dL, Kruskall–Wallis test, *p* < 0.001) in leptin-treated fasted animals (Fig. 2C).

We also observed reduced plasma glucose levels (fasted + saline = 102 ± 6.1 mg/dL, fasted + 0.5 leptin = 66.3 ± 3.3 mg/dL and fasted + 1.0 leptin = 67.8 ± 3.4 mg/dL, Kruskall–Wallis test, p < 0.001) in leptin-treated fasted animals (Fig. 2C). Administration of leptin to fasted animals increased the mRNA expression of Pomc (fasted + saline = 0.9 ± 0.1 AU, fasted + 0.5 leptin = 1.4 ± 0.1 AU and fasted + 1.0 leptin = 1.4 ± 0.2 AU, one-way ANOVA followed by post hoc Tukey’s multiple comparison, p < 0.05) and reduced Npy (fasted + saline = 1.9 ± 0.2 AU, fasted + 0.5 leptin = 1.7 ± 0.2 AU and fasted + 1.0 leptin = 1.1 ± 0.0 AU, one-way ANOVA followed by post hoc Tukey’s multiple comparison, p < 0.05) and Gad67 (fasted + saline = 1.3 ± 0.0 AU, fasted + 0.5 leptin = 0.9 ± 0.0 AU and fasted + 1.0 leptin = 0.9 ± 0.0 AU, one-way ANOVA followed by post hoc Tukey’s multiple comparison, p < 0.05) in the ARC (Fig. 2D).

### Chemogenetic activation of POMC^ARC^ neurons do not affect fasting-induced HPA axis activation

**Fig. 3:**
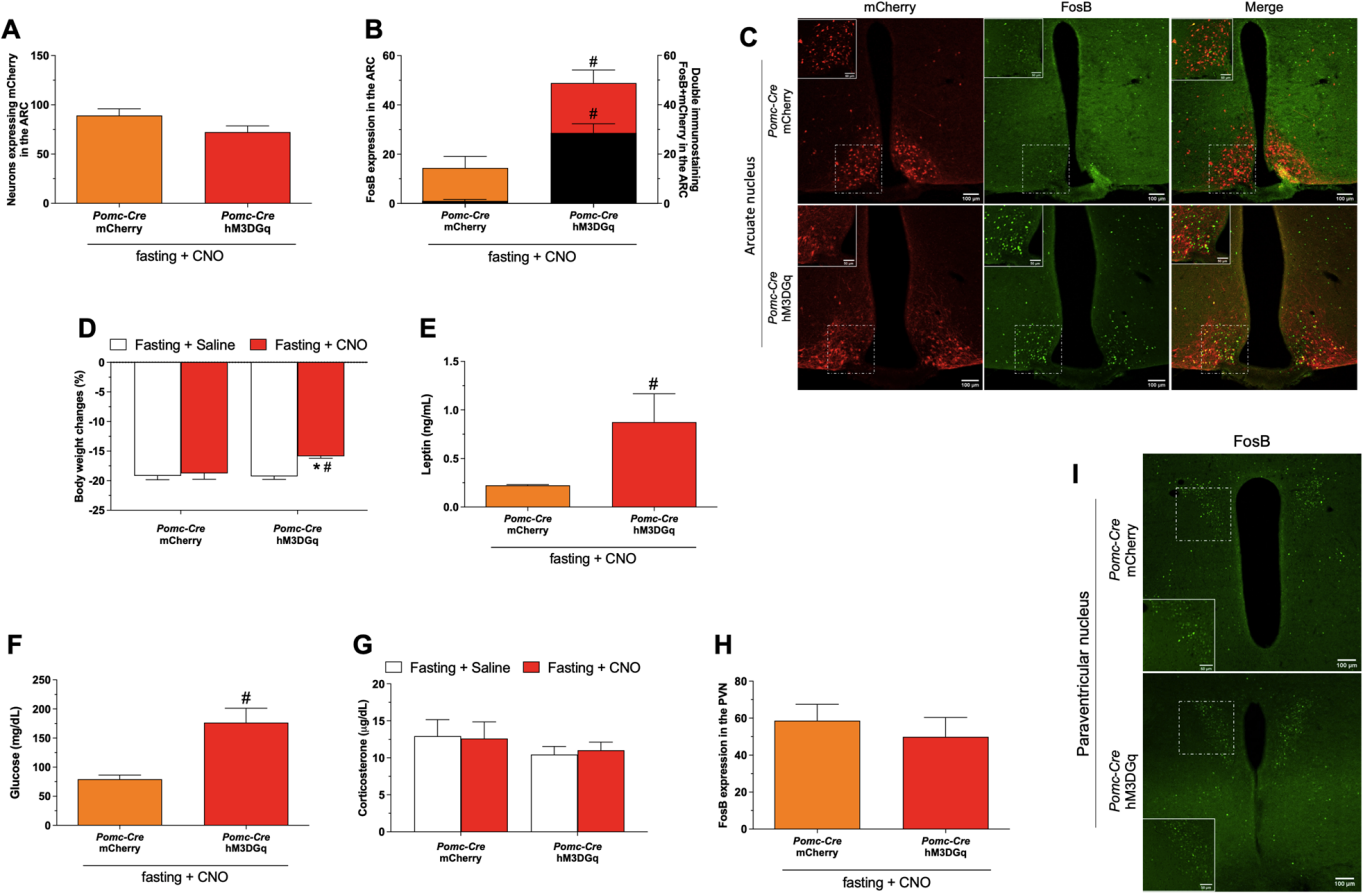
Chemogenetic activation of POMC^ARC^ neurons do not modulate fasting-induced HPA axis activation: Number of mCherry positive neurons (**A**), FosB (left y scale and color bars) and double mCherry/FosB (right y scale and black bars) expression in the ARC (**B**), representative photomicrographs (×10 and insert ×20) of double immunostaining mCherry/FosB in the ARC (**C**), body weight change (**D**), plasma leptin (**E**), glucose (**F**) and corticosterone (**G**) levels, PVN FosB expressing neurons (**H**) and representative photomicrographs (×10 and insert ×40) of FosB in the PVN (**I**) of adult (8–12 weeks) male *Pomc-Cre* mice (n = 9–18) that received intra ARC injection of AAV-DIO-mCherry or AAV-DIO-hM3D(Gq)-mCherry submitted to 36 h of fasting and treated with 5 i.p. injections CNO (1 mg/kg) or saline. The i.p. injections were performed each 8 h and the animals were euthanized at 10am as described in the Additional file 1: Fig. S1. Data are expressed as mean ± SEM and were analyzed by two-way ANOVA followed by post hoc Tukey’s multiple comparison (**D** and **G**) or unpaired t test or Mann–Whitney (**A**, **B**, **E**, **F** and **H**). *p < 0.05 *vs* saline; ^#^*p* < 0.05 *vs Pomc-Cre::*AAV-DIO-mCherry

Bilateral AAV-DIO-hM3DGq-mCherry and AAV-DIO-mCherry injections in the ARC were equally efficient in targeting POMC^ARC^ neurons (Fig. 3A, mCherry = 89.2 ± 6.8 mCherry + cells and hM3DGq = 72.3 ± 6.2 mCherry + cells, unpaired *t* test, *p* = 0.1). We confirmed the chemogenetic activation of POMC^ARC^ neurons by the increased FosB + mCherry expression in the ARC (mCherry = 1.0 ± 0.6 FosB + mCherry cells and hM3DGq = 28.6 ± 3.7 FosB + mCherry cells, Mann–Whitney test, *p* = 0.002) of *Pomc-Cre* animals treated with CNO (Fig. 3B,C). Surprisingly, activation of POMC^ARC^ reduced fasting-induced body weight loss (Fig. 3D, mCherry + saline = − 19.2 ± 0.6%BWC, mCherry + CNO = − 18.8 ± 1.0%BWC, hM3DGq + saline = -19.3 ± 0.6%BWC and hM3DGq + CNO = − 15.8 ± 0.3%BWC, two-way ANOVA followed by post hoc Tukey’s multiple comparison, * and ^#^*p* = 0.01) and this response was associated with increased plasma leptin (Fig. 3E, mCherry = 0.2 ± 0.0 ng/mL and hM3DGq = 0.9 ± 0.3 ng/mL, Mann–Whitney test, *p* = 0.01) and glucose levels (Fig. 3F, mCherry = 79.3 ± 7.1 mg/dL and hM3DGq = 176.6 ± 24.8 mg/dL, Mann–Whitney test, *p* = 0.001).

Surprisingly, activation of POMCARC reduced fasting-induced body weight loss (Fig. 3D, mCherry + saline = − 19.2 ± 0.6%BWC, mCherry + CNO = − 18.8 ± 1.0%BWC, hM3DGq + saline = -19.3 ± 0.6%BWC and hM3DGq + CNO = − 15.8 ± 0.3%BWC, two-way ANOVA followed by post hoc Tukey’s multiple comparison, * and #p = 0.01) and this response was associated with increased plasma leptin (Fig. 3E, mCherry = 0.2 ± 0.0 ng/mL and hM3DGq = 0.9 ± 0.3 ng/mL, Mann–Whitney test, p = 0.01) and glucose levels (Fig. 3F, mCherry = 79.3 ± 7.1 mg/dL and hM3DGq = 176.6 ± 24.8 mg/dL, Mann–Whitney test, p = 0.001). In addition, the specific activation of POMC_ARC_ had no effect on the increased plasma corticosterone levels in fasted animals (Fig. 3G, mCherry + saline = 12.9 ± 2.2 μg/dL, mCherry + CNO = 12.6 ± 2.2 μg/dL, hM3DGq + saline = − 10.4 ± 1.1 μg/dL and hM3DGq + CNO = − 11.0 ± 1.1 μg/dL, two-way ANOVA followed by post hoc Tukey’s multiple comparison). Fasting-induced FosB expression in PVN neurons was not affected by activation of POMC_ARC_ neurons (Fig. 3H,I mCherry = 58.6 ± 8.9 FosB + cells and hM3DGq = 49.9 ± 10.4 FosB + cells, Mann–Whitney test, p = 0.5). Similar results to these described here were observed comparing Pomc-Cre and WT animals treated with AAV-DIO-hM3DGq-mCherry (Additional file 3: Fig. S3).

### Chemogenetic inhibition of AgRP^ARC^ neurons reduces PVN neuronal activity without affecting plasma corticosterone levels in fasted animals

**Fig. 4:**
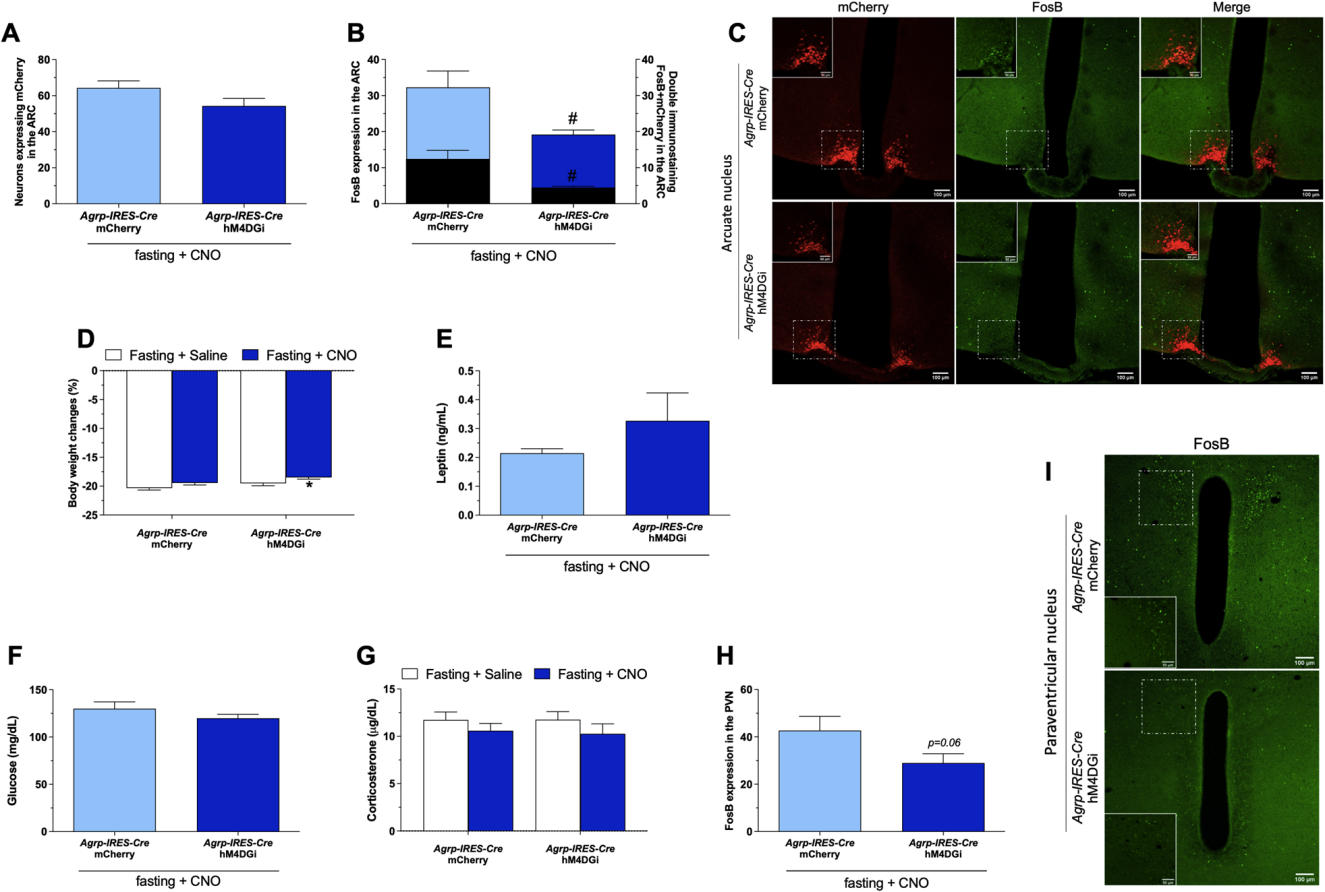
Chemogenetic inhibition of AgRP^ARC^ neurons reduces PVN neuronal activity without affecting plasma corticosterone levels in fasted animals: Number of mCherry positive neurons (**A**), FosB (left y scale and color bars) and double mCherry/FosB (right y scale and black bars) expression in the ARC (**B**), representative photomicrographs (×10 and insert ×20) of double immunostaining mCherry/FosB in the ARC (**C**), body weight change (**D**), plasma leptin (**E**), glucose (**F**) and corticosterone (**G**) levels, PVN FosB expressing neurons (**H**) and representative photomicrographs (×10 and insert ×40) of FosB in the PVN (**I**) of adult (8–12 weeks) male *Agrp-IRES-Cre* mice (n = 14–18) that received intra ARC injection of AAV-DIO-mCherry or AAV-DIO-hM4D(Gi)-mCherry submitted to 36 h of fasting and treated with 5 i.p. injections CNO (1 mg/kg) or saline. The i.p. injections were performed each 8 h and the animals were euthanized at 10am as described in the Additional file 1: Fig. S1. Data are expressed as mean ± SEM and were analyzed by two-way ANOVA followed by post hoc Tukey’s multiple comparison (**D** and **G**) or unpaired t test or Mann–Whitney (**A**, **B**, **E**, **F** and **H**). *p < 0.05 *vs* saline; ^#^*p* < 0.05 *vs Agrp-IRES-Cre::*AAV-DIO-mCherry

We observed similar mCherry expression after bilateral injections of AAV-DIO-hM4DGi and AAV-DIO-mCherry in *AgrpIRES-Cre* animals (Fig. 4A, mCherry = 64.3 ± 3.9 mCherry + ells and hM4DGi = 54.3 ± 4.1 mCherry + cells, unpaired *t* test, *p* = 0.1). CNO treatment reduced FosB + mCherry colocalization (Fig. 4B, mCherry = 12.4 ± 2.4 FosB + mCherry cells and hM4DGi = 4.4 ± 0.4 FosB + mCherry cells, Mann–Whitney test, *p* = 0.003) in AgRP^ARC^ neurons in fasted animals expressing hM4DGi (Fig. 4B,C). The specific inhibition of AgRP^ARC^ neurons reduced the activation of PVN neurons (Fig. 4H, I, mCherry = 42.7 ± 6.0 FosB +  cells and hM4DGi =  29 ±  3.9 FosB +  cells, unpaired *t* test, *p* = 0.0687) but had no effect on fasting-induced corticosterone secretion (Fig. 4G, mCherry +  saline =  11.7 ±  0.8 µg/dL, mCherry +  CNO =  10.6 ±  0.8 µg/dL, hM4DGi +  saline =  11.7 ±  0.8 µg/dL and hM4DGi +  CNO =  10.3 ±  1.0 µg/dL, two-way ANOVA followed by post hoc Tukey’s multiple comparison).

We also observed reduced body weight loss (Fig. 4D, mCherry + saline = − 20.3 ± 0.3%BWC, mCherry + CNO = − 19.4 ± 0.3%BWC, hM4DGi + saline = − 19.5 ± 0.4%BWC and hM4DGi + CNO = − 18.5 ± 0.3%BWC, twoway ANOVA followed by post hoc Tukey’s multiple comparison, *p = 0.003) after activation of AgRPARC neurons and no difference in the reduction of plasma leptin (Fig. 4E, mCherry = 0.2 ± 0.0 ng/mL and hM4DGi = 0.3 ± 0.1 ng/mL, Mann– Whitney test, p = 0.5) and glucose (Fig. 4F, mCherry = 130 ± 7.1 mg/dL and hM4DGi = 119.8 ± 4.2 mg/dL, Mann–Whitney test, p = 0.3) levels between the groups. Similar results to these described here were obtained comparing Agrp-IRES-Cre and WT animals that received icv injection of AAV-DIO-hM4DGi-mCherry (Additional file 4: Fig. S4).

### Inhibition of AgRP^ARC^ neurons in fasted animals reduces CRF content in the PVN and increases its accumulation in the median eminence

**Fig. 5:**
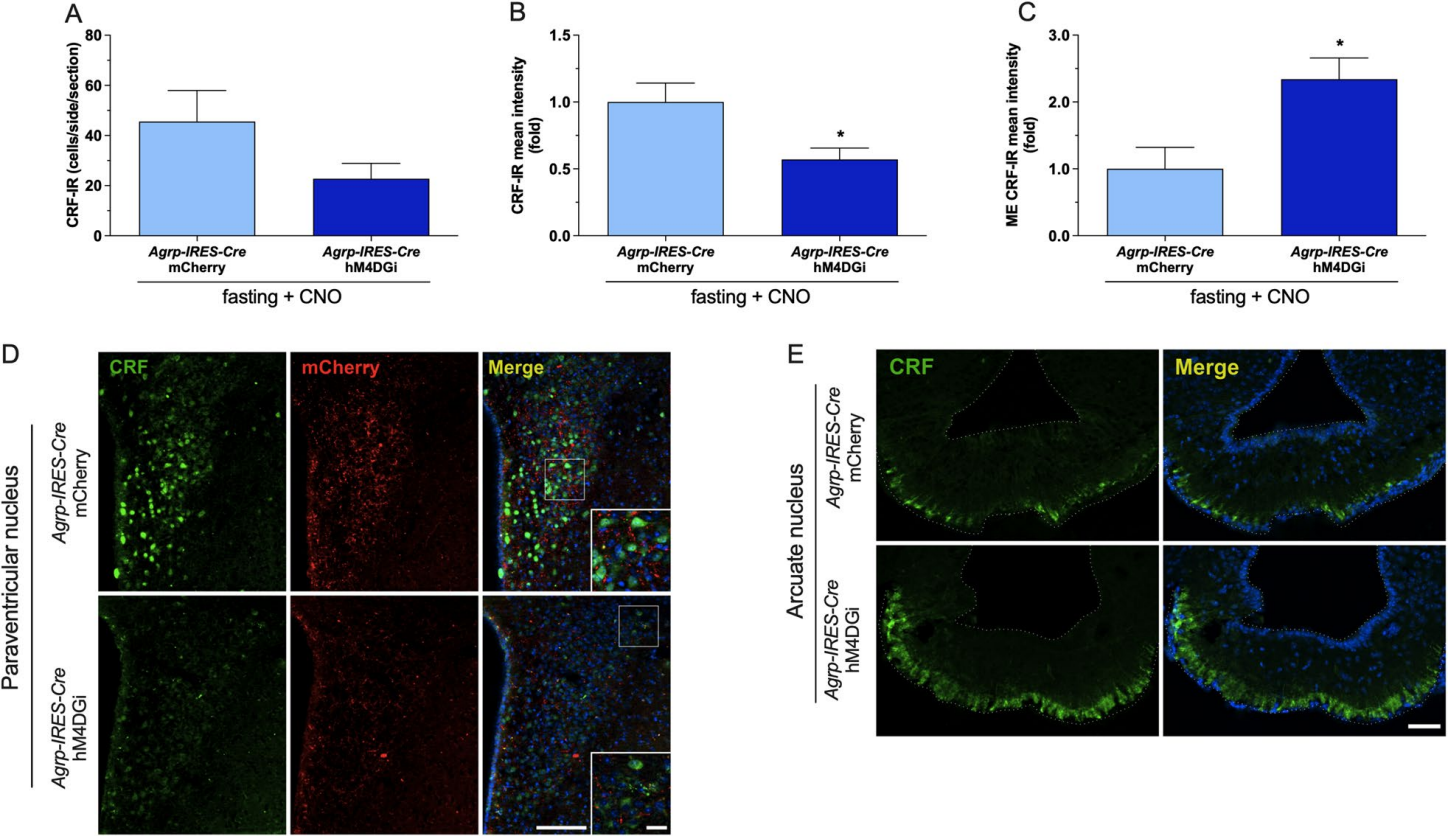
Inhibition of AgRP^ARC^ neurons in fasted animals reduces CRF content in the PVN and increases its accumulation in the median eminence: Representative photomicrographs (**D** and **E**) and quantitative CRF-IR (green) cell number (**A**) and mean intensity (**B** and **C**) in both PVN and median eminence of *Agrp-IRES-Cre* animals that received intra ARC injection of AAV-DIO-mCherry or AAV-DIO-hM4D(Gi)-mCherry (red) submitted to 36 h of fasting and treated 5 i.p. injections CNO (1 mg/kg). Tissue samples obtained from experimental procedure described in the Fig. 3 were used for CRF immunostaining studies. The cell nuclei were staining with Hoechst (blue). Data are expressed as mean ± SEM and were analyzed by unpaired t test: *p < 0.05 *vs Agrp-IRES-Cre::* AAV-DIO-mCherry fasted and treated with CNO

In our CRF immunostaning study, we observed reduced CRF-IR in the PVN (Fig. 5B,D, mCherry = 169.8 ± 24 and hM4DGi = 96.8 ± 14.5, unpaired *t* test, *p* = 0.02), whereas the level of this peptide increased in the ME (Fig. 5C,E, mCherry = 45.2 ± 14.5 CRF-IR and hM4DGi = 105.8 ± 14.3 CRF-IR, unpaired *t* test, *p* = 0.001) in *Agrp-IRES-Cre* animals expressing hM4DGi and treated with CNO. However, we observed no difference in the number of CRF + cells (Fig. 5A,D, mCherry = 45.6 ± 12.4 and hM4DGi = 22.7 ± 6.2, unpaired *t* test) in the PVN. We also observed similar results comparing *Agrp-IRES-Cre* and WT animals treated with AAV-DIO-hM4DGi-mCherry (Additional file 5: Fig. S5).

## Discussion

In the present study we demonstrated the effects of food deprivation on the HPA axis activity and the involvment of AgRP^ARC^ neurons in the gene expression/activation of CRF^PVN^ neurons, the major HPA axis controller.

It’s well know that fasting activates HPA axis [9]. Here we demonstrated that the increased plasma corticosterone levels observed in fasted mice was associated to increased mRNA expression and activity of CRF^PVN^. The increased plasma glucocorticoid levels in fasted animals and humans are essential to the energy substrate utilization (fatty acids instead of carbohydrates) and to the activation of hepatic gluconeogenesis to avoid hypoglycemia [22, 23]. Interestingly, it has recently been demonstrated that hyperphagia caused by various energy-depleted states, such as food deprivation, hypoglycemia, or diabetes, is also dependent on increased plasma corticosterone levels and its actions on arcuate neurons controlling food consumption [24].

The reduction in body weight and the consequent decrease in plasma glucose and leptin levels in mice fasted for 36 h can contribute to the changes in the HPA axis activity [9–11]. Indeed, fasting-induced hypoglycemia can recruit the counter-regulatory response and consequently increase glucagon, epinephrine and corticosterone secretion [25, 26]. Additionally, the decrease in the syhthesis of leptin from the white adipose depots [27] leading to a reduction of plasma leptin levels is required for metabolic adaptations to reduce energy availability and activation of the counter-regulatory response [25].

Regarding the mRNA expression in the ARC, we observed increased mRNA expression of *Agrp, Npy* and *Gad65/67* in the ARC, and decreased *Pomc* mRNA expression in mice fasted for 36 h. These data reinforce the role and the responsivity of neuropeptides/neurotransmitters expressed in ARC neurons in the control of energy homeostasis [7, 8, 10]. Together, our data indicate that a prolonged fasting period changes the mRNA expression and activity of CRF^PVN^ neurons in response to changes in ARC mRNA expression/activity of neuropeptides/neurotransmitters projecting to PVN.

Leptin can reduce or increase the HPA axis activity and its response it seems to be associated with the amount of energy available, i.e. in fasted animals the most prevalent response observed in scientific studies is a decrease in plasma corticosterone levels after leptin administration [9, 11, 28]. Furthermore, even during food deprivation, this effect of leptin appears to be dependent on the level of the reduction of energy storage, as increased HPA activity was reported in studies using a prolonged period of fasting (e.g. 48 h) [9, 24] but not in 24 h fasted animals [24, 29]. In our study, we observed increased body weight loss and plasma corticosterone levels in animals fasted for 24 h, but higher body weight loss and plasma corticosterone levels after 36 h of fasting (Additional file 2: Fig. S2). The reduced mRNA expression of CRF^PVN^ and neuronal activity of CRF^PVN^ neurons are in accordance with the inhibitory role of leptin on the HPA axis activity [9, 28].

The effects of leptin on the CRF gene expression and CRF^PVN^ neuronal activity can not be mediated by direct leptin signaling, since LepR is not expressed in CRF^PVN^ neurons [13]. Additionally, ARC neurons have their gene expression (Fig. 2D) and activity modulated by leptin [12, 14] and, as we previously cited, icv or even site-specific administration of the neuropeptides/neurotransmiters produced by ARC modulates the HPA axis activity [30, 31]. Therefore, the changes in the ARC mRNA could account for the reduction in the HPA axis activity after leptin administration. In addition, the decrease of plasma corticosterone levels in fasted mice under leptin treatment can contribute to the reduction of plasma glucose levels. However, leptin may also affect other key glucoregulatory mechanisms (i.e., sympathetic nervous system activity) [22, 32–34] to modulate plasma glucose levels. Together, our data reinforce the inhibitory role of leptin in the control of CRF^PVN^. Furthermore, a direct leptin signaling on adrenal gland [35] and/or indirect actions via ARC neurons could also be involved in the reduction of plasma corticosterone levels after leptin treatment to fasted animals.

POMC^ARC^ neurons project to the PVN [17] and are modulated by fasting [36] and leptin [14]. Thus, we investigated the consequences of the chemogenetic activation of POMC^ARC^ on plasma corticosterone levels in 36 h-fasted mice. Despite the effective chemogenetic activation of POMC^ARC^ neurons in fasted animals expressing the excitatory DREADD, we observed no effect on PVN neuronal activity and on the increased plasma corticosterone levels induced by food deprivation. POMC^ARC^ neurons are heterogenous regarding their responsivity to hormones such as leptin [37] and particularly regarding to the type of the neurotransmitter they express (e.g., AgRP, NPY and GABA) [38, 39]. Therefore, the heterogeneity of these neurons must be considered before definitely excluding their participation in the control of the HPA axis activity in fasting conditions. A study using the same lineage of animals reported that POMC neurons projects to the adrenal gland, and the deletion of LepR in this neuronal population does not modify the activation of SNS induced by icv leptin [40]. We cannot exclude the possibility that the development of compensatory mechanism during development could have affected the evaluation of the role of POMC^ARC^ neurons in the effects of leptin on ANS in the Bell and cols study [40]. Thus, our data demonstrated that the prolonged activation of the heterogenous population of POMC^ARC^ neurons do not modify the corticosterone plasma levels during fasting.

Additionally, activation of POMC^ARC^ reduced fasting-induced body weight loss and increased plasma leptin and glucose levels. Notably, only one-third of CNO-treated *Pomc-Cre*::hM3DGq mice had plasma leptin levels higher than the control group suggesting that the effect of prolonged activation of POMC^ARC^ neurons on plasma leptin levels was secondary to body weight loss instead of a direct effect of POMC^ARC^ neuronal modulation, as described by others [41]. Our data about body weight change and plasma glucose levels in fasted animals subjected to prolonged POMC^ARC^ activation are contradictory to the classical role of these neurons on energy and glucose homeostasis. Despite some studies have observed the expected effect on energy balance after chemogenetic modulation of POMC^ARC^ neurons (i.e. reduced food intake after activation) [42, 43], there are divergent results about its effect on glucose homeostasis. So, it was reported that the selective activation of POMC^ARC^ neurons can reduce pyruvate-induced hyperglycemia [44], while other study described no effect on plasma glucose levels after POMC^ARC^ modulation [45] or even a fall in glycemia after inhibition of POMC^ARC^ neurons [42]. Therefore, our data support the heterogeneity of these neurons and suggests that the responses observed in our study are involved with the activation of a subset of POMC^ARC^ neuron that could also produce AgRP, NPY and/or GABA that could inhibit energy metabolism.

Given that the food consumpition-effects induced by activation of AgRP^ARC^ neurons are mediated, at least in part, by modulating the activity of PVN neurons [46–48] and that these ARC neurons are inhibited by leptin [10] we also investigated whether the specific inhibition of AgRP^ARC^ neuronal activity could modulate plasma corticosterone levels in fasted animals. Our findings show that inhibiting AgRP^ARC^ neurons has no effect on fasting-increased plasma corticosterone levels, but it is effective in reducing PVN neuron activity. The lack of changes in corticosterone secretion after inhibiting of AgRP^ARC^ neuronal activity is opposite to the results reported by pharmacological studies, which observed increased corticosterone secretion after intra PVN administration of NPY [15, 16], the neuropeptide coexpressed with AgRP^ARC^. On the other hand, the present data on FosB study indicates that specific inhibition of AgRP^ARC^ neurons reduces the activity of PVN neurons. Therefore, according to an excitatory role of NPY on PVN neurons, it was described that the NPYr2 can be coupled to Gq signaling [49]. Therefore, inhibition of AgRP^ARC^ neurons could reduce NPY signaling via NPYr2 expressed in the PVN that in turn could lead to a decreased PVN neuronal activity in fasted conditions. However, we cannot exclude an indirect effect of AgRP^ARC^ neurons on the PVN activity, since AgRP^ARC^ neurons project to other brain sites that in turn are directly connected to PVN neurons [17, 18].

Recently, it was demonstrated that GABAergic neurons from ARC are directly connected to CRF^PVN^ neurons, supporting the involvement of this ARC neurotransmitter in the control of the HPA axis [18]. Interestingly, under prolonged hyperosmotic stress, GABAergic signaling in the PVN is switched from inhibitory to excitatory [50, 51]. Moreover, administration of GABA_A_R agonist can induce CRF releasing in the median eminence [52]. Therefore, the inhibition of NPY and GABA release from AgRP^ARC^ neurons could also contribute to the reduced neuronal activity in the PVN during fasting.

As expected inhibition of AgRP^ARC^ reduced fasting-induced body weight loss, but didn’t change the effects of fasting on plasma leptin and glucose levels. AgRP^ARC^ neurons are known to negatively modulate the metabolism and part of these responses can be associated with a change in the thyroid axis activity and energy expenditure [53]. Therefore, reduction in energy expenditure after inhibition of AgRP^ARC^ can be associated with the reduced body weight loss.

Since we had observed reduced PVN neuronal activity after inhibition of AgRP^ARC^ neurons in fasted animals, we also investigated the content of CRF in the PVN and its releasing in the median eminence in these animals. Interestingly, our data indicates AgRP^ARC^ neurons can modulate the synthesis and secretion of CRF^PVN^ during metabolic stress. Our results corroborate the data from Kakizawa and Cols that observed increased CRF release in the ME after administration of GABA_A_R agonist [52]. Therefore, our findings are consistent with other studies indicating an excitatory role of AgRP^ARC^ neurons on CRF^PVN^ expression/activity [15, 51, 52, 54] and highlight AgRP^ARC^ neurons as a component of the neurocircuitry controlling the activity of CRF^PVN^ neurons during fasting.

Despite the reduced CRF content in the medial parvocellular PVN subdivision and its accumulation in the ME, plasma corticosterone levels were not reduced in *Agrp-IRES-Cre* mice expressing hM4DGi treated with CNO. To conciliate these results, we need to consider the involvement of different pathways an mechanisms that underlie stress-induced increase in plasma corticosterone levels. So, considering that AgRP^ARC^ neuronal inhibition induced a slight fall in PVN FosB expression, other PVN peptides involved in the control of pituitary-ACTH activity and ANS, such as vasopressin and even CRF expressed in PVN autonomic neurons, could act to maintain the increased plasma corticosterone levels via modulation of adrenal gland activity (neural mechanisms or dependent of ACTH) [55–58]. Additionally, mechanisms associated with changes in the sensitivity to the ACTH [59] and/or peripheral glucocorticoid metabolism [60, 61] may contribute to the preserved corticosterone secretion in *Agrp-IRES-Cre*::hM4DGi mice treated with CNO. Therefore, the existence of redundant pathways/neurocircuitries and mechanisms involved in the control of HPA axis are probably underlying the maintenance of increased plasma corticosterone levels in fasted animals after the selective inhibition of AgRP^ARC^ neurons.

Finally, since CRF^PVN^ neurons are involved in the control of endocrine, autonomic, and behavioral responses to stress [2–4], we speculate that its reduced content in the PVN with the inhibition of AgRP^ARC^ neurons could be related to the modulation of other stress-related responses that were not investigated in our study. Given that AgRP^ARC^ neurons modulate several motivational components of food consumption [54] and that CRF^PVN^ also controls several behavioral stress-related responses [4], part of those behavioral changes elicited by AgRP^ARC^ neurons could be associated to change in the activity of CRF^PVN^ neurons.

## Conclusion

Our study reinforces the recruitment of HPA axis during fasting and the involvement of leptin in this response. Additionally, we demonstrate that AgRP^ARC^, but not POMC^ARC^, are part of the neurocircuitry involved in the control of CRF^PVN^ neurons activity during fasting. The uncovering of the mechanism and neurocircuitry involved in the coupling of HPA axis activity to changes in peripheral energy stores could be considered an important strategy to the development of new therapies to treat of pathologies associated with the disturbance of the HPA axis, including obesity and metabolic syndrome.

## Methods

### Animals

All procedures were approved by the Ethical Committee for Animal Use of the University of Ribeirao Preto (12/2017, February 2018) and Federal University of Sao Paulo (07/2020-1276210720). C57BL6 male mice were obtained from Central Animal Facility of the University of Sao Paulo-Campus Ribeirao Preto. Male *Agrp-IRES-Cre*, *Pomc-Cre* mice, *Crh-IRES-Cre* and the Cre-inducible tdTomato-reporter mouse were purchased from JAX mice (Bar Harbor, ME) and genotyped as previously described [62, 63]. The animals were housed in groups under controlled light (12:12 h light–dark cycle; lights off at 06:00 p.m.) and temperature conditions (23 ± 2 °C), with free access to water and food, unless otherwise stated.

### Experimental procedures

For all protocols, mice were single housed for at least 1 week before the beginning of the experiments and habituated to researcher manipulation. Flow charts of the experimental procedures are described in Additional file 1: Fig. S1.

For the fasting study, mice were weighed and maintained on regular diet or fasted for 36 h. At the end of the experiment (10 a.m.) they were weighed again and immediately decapitated for blood (heparinized tube) and tissue collection. In the leptin treatment experiment, fasted mice were treated with 4 i.p. injections of saline or leptin [Millipore (Burlington, MA)—doses of 0.5 µg/g or 1 µg/g)] at 6am, 2 pm, 10 p.m. of day 2 and at 6am on day 3 (last day). At 10 a.m. fasted mice treated with saline or leptin were weighed again and decapitated for blood and tissue collection. Perfusion for immunostaining studies (described below) were performed using *Crh-IRES-Cre*:tdTomato-reporter mouse.

For DREADDs experiments, 2 weeks after surgery, WT, *Pomc-Cre* or *Agrp-IRES-Cre* mice were fasted and treated with 5 i.p. injections of saline + 1% DMSO (10 p.m.–day1, 6 a.m., 2 p.m., 10 p.m.–day2 and 6 a.m.–day3) and their blood collected via submandibular bleed. After 2 weeks the same mice were fasted again and treated with 5 i.p. injections of Clozapine-N-Oxide [Cayman Chemical (Ann Arbor, MI)—1 mg/kg] (10 p.m.–day1, 6 a.m., 2 p.m., 10 p.m.–day2 and 6 a.m.–day3) and then quickly anesthetized with isoflurane and perfused for blood and brain collection.

### Viral vectors microinjection and stereotaxic surgery

Anesthetized mice were submitted to stereotaxic surgery for Adeno-associated viral (AAV) injection [64]. A volume of 200 nL of pAAV8-hSyn-DIO-hM3D(Gq)-mCherry, pAAV8-hSyn-DIO-hM4D(Gi)-mCherry or pAAV8-hSyn-mCherry (Addgene, Watertown, MA) was bilaterally injected into the ARC (AP: − 1.4, DV: − 5.85 and ML: ± 0.3 from bregma) during 5 min (40 nL/min); the pipette was removed 5 min after injection. The AAVs contain either the excitatory or inhibitory DREADD and the mCherry sequence in reverse orientation and, flanked by loxP sites. The presence of Cre-recombinase flips the DNA so that it can be transcriptionally expressed. We used mice that express Cre-recombinase in either AgRP/NPY neurons (*Agrp-IRES-Cre* mice) or POMC neurons (*Pomc-cre* mice). We also want to mention that a subset of POMC neurons colocalize with NPY/AgRP during embryonic development [38, 39].

After the surgery, mice had 2 weeks to recover from the surgical procedure and to acclimate to be individually housed before the beginning of the experiments. The 2 weeks period was also necessary for the expression of the DREADDs. To confirm the correct injection and expression of the DREADDs, mCherry expression was visualized in the ARC.

### Transcardiac perfusion, tissue collection and immunostaining

Mice were perfused and the tissue processed to obtain 25-µm coronal sections as previously described [40, 42, 46]. Briefly, to perform FosB immunostaining, brain sections were rinsed with Phosphate Buffered Saline (PBS; 1×, pH 7.4), pre-treated for 1 h in PBS containing 5% normal donkey serum (Jackson ImmunoResearch Laboratories, Inc., West Grove, PA, USA) and 0.3% Triton X-100 and then incubated with a rabbit anti-FosB polyclonal IgG (1:1000, Santa Cruz Biotechnology, #sc48) overnight at room temperature. After rinsing, sections were incubated with a donkey anti-rabbit IgG conjugated with Alexa 488 secondary antibody (1:400; Jackson ImmunoResearch Laboratories, Inc., West Grove, PA, USA). The mCherry fluorescence could be observed without immunostaining. Finally, the sections were coverslipped with Fluoromont-G mounting medium (Sigma-Aldrich, USA).

We performed CRF immunofluoresce studies as described before [65]. Briefly, sections were pre-treated with blocking solution (2% normal horse serum and 0.25% Triton-X) and then incubated with a rabbit anti-CRH antibody (1:1000) for 48 h at 4 °C. This antibody recognizes mature CRF (1–41) and full pre-proCRF prohormone [66]. Next, sections were incubated with a goat anti-rabbit Alexa Fluor 488 antibody (1:1000, Thermo Fisher, Waltham, MA) for 2 h. Brain sections were sequentially mounted on glass slides and coverslipped with mounting media.

### Quantitative neuroanatomical analysis

A Zeiss inverted Fluorescence motorized phase contrast microscope was used to acquire the photomicrographs associated to FosB data. Two or three images from each animal were quantified for mCherry, FosB only or double FosB + mCherry using Image J software (NIH, Bethesda, MD). Only animals mCherry + in both sides of the ARC were used in the experiments. We used mCherry as marker of specificity and efficiency of viral vector transduction and FosB as a marker of prolonged neuronal activity. FosB immunofluoresce + tdTomato (reporter animals) were used to indicate changes in CRF^PVN^ neuronal activity.

For CRF-IR studies, fluorescence images were acquired with 20×/0.80 and 40×/0.95 objectives using a Zeiss AxioObserver D1 equipped with an Apotome.2 structured illumination module and an AxioCam 506 monochrome camera. All image processing and analysis were performed in the ImageJ-based open-source image-processing package Fiji (NIH, Bethesda, MD) [67]. Fluorescent signal corresponding to CRH + was blindly and bilaterally quantified in the PVN and EM. The average fluorescence intensity was quantified in low magnification (20×) images of one complete series of coronal sections per brain between bregma − 0.58 and − 0.94 mm for the PVN and between bregma − 1.58 and − 1.94 mm for the EM [68]. For each image, the histogram of signal intensity was obtained, and the tissue background level was estimated by fitting a Gaussian curve, which coincided with the dominant peak. With these parameters, a specific signal detection threshold was calculated, which was defined as the mean of the distribution plus five standard deviations. A region of interest was created according to this threshold. The mean fluorescence intensity was quantified in each image within this thresholded region.

### Brain microdissection, RNA isolation and qPCR

The PVN and ARC microdissections were performed with a stainless-steel punch needle (1.0 mm diameter) in a cryostat according to the coordinates from − 0.18 to − 1.055 mm (~ 800 µm) and from − 1.055 to − 2.255 mm (1200 µm) bregma, respectively [68].

Total RNA from PVN and ARC were extracted using RNeasy Mini Kit (QIAGEN, Hilden, Germany) and reverse transcribed (QuantiTect RT Kit, QIAGEN). For TaqMan gene expression assay, qPCR master mix QuantiNova kit (QIAGEN) and specific primers from Integrated DNA Technology (IDT, Coralville, IA) were used. Quantitative PCR was performed in triplicate using the QuantStudio 5 (Applied Biosystems). Relative mRNA levels were calculated using the standard curve method and normalized to the level of b-actin.

### Plasma hormones and glucose determinations

Plasma corticosterone levels were determined by specific radioimmunoassays after extraction with ethanol, as previously described [69]. Plasma leptin (Millipore, Burlington, MA) and glucose (Doles Reagents, Goiania, Brazil) concentrations were measured using commercial kits.

### Statistical analysis

The data are expressed as mean ± SEM. Samples were tested for regular distribution using Shapiro–Wilk normality test (Prism9.0-GraphPad software, San Diego, CA). We used one- or two-way ANOVA, followed by the appropriate post hoc tests, or an unpaired Student's t test (or the Mann–Whitney test for non-parameteric data). Differences were accepted as significant if p < 0.05. The specific statistical tests that were used are indicated in the figure legends.

## Supplementary Information

**Additional file 1: Figure S1.** Experimental designs used in the protocols performed to obtain data showed in Fig. 1 (fasting and fasting + leptin studies) and Figs. 2, 3 and 4.

**Additional file 2: Figure S2.** Prolonged fasting increases *Crf* mRNA expression in the PVN and induces higher corticosterone secretion: Experimental design (A), body weight change (B), plasma leptin (C), glucose (D) and corticosterone (E) levels, changes in the mRNA expression in the PVN (F) and ARC (G) of adult (8–10 weeks) WT mice fed or fasted for 24 h or 36 h (n = 8–10). Data are expressed as mean ± SEM and were analyzed by one-way ANOVA and Tukey test for multiple comparisons were used for samples that passed in the normality test (B, C, D and E) otherwise Kruskal–Wallis test followed by Dunn’s multiple comparisons were used (F and G): *p < 0.05 *vs* fed and ^#^p < 0.05 *vs* 24 h fasting.

**Additional file 3: Figure S3.** Chemogenetic activation of POMC^ARC^ neurons do not modulate fasting-induced HPA axis activation: FosB expression in the ARC (A), representative photomicrographs (10× and insert 20×) of double immunostaining mCherry/FosB in the ARC (C), body weight change (D), plasma leptin (E), glucose (F) and corticosterone (G) levels, PVN FosB expressing neurons (H) and representative photomicrographs (10× and insert 40×) of FosB in the PVN (I) of adult (8–12 weeks) male WT or *Pomc-Cre* mice (n = 8–18) that received intra ARC injection AAV-DIO-hM3D(Gq)-mCherry submitted to 36 h of fasting and treated with 5 i.p. injections CNO (1 mg/kg) or saline. The i.p. injections were performed each 8 h and the animals were euthanized at 10am as described in the Additional file 1: Fig. S1. Data are expressed as mean ± SEM and were analyzed by two-way ANOVA followed by post hoc Tukey’s multiple comparison (C and F) or unpaired t test. *p < 0.05 *vs* saline; ^#^*p* < 0.05 *vs Pomc-Cre::*AAV-DIO-mCherry.

**Additional file 4: Figure S4.** Chemogenetic inhibition of AgRP^ARC^ neurons reduces PVN neuronal activity without affecting plasma corticosterone levels in fasted animals: FosB expression in the ARC (A), representative photomicrographs (10× and insert 20×) of double immunostaining mCherry/FosB in the ARC (C), body weight change (D), plasma leptin (E), glucose (F) and corticosterone (G) levels, PVN FosB expressing neurons (H) and representative photomicrographs (10× and insert 40×) of FosB in the PVN (I) of adult (8–12 weeks) male WT or *Agrp-IRES-Cre* mice (n = 8–18) that received intra ARC injection AAV-DIO-hM4D(Gi)-mCherry submitted to 36 h of fasting and treated with 5 i.p. injections CNO (1 mg/kg) or saline. The i.p. injections were performed each 8 h and the animals were euthanized at 10am as described in the Additional file 1: Fig. S1. Data are expressed as mean ± SEM and were analyzed by two-way ANOVA followed by post hoc Tukey’s multiple comparison (C and F) or unpaired t test. *p < 0.05 *vs* saline; ^#^*p* < 0.05 *vs Agrp-IRES-Cre::*AAV-DIO-mCherry.

**Additional file 5: Figure S5.** Inhibition of AgRP^ARC^ neurons reduces PVN CRF content and induces its accumulation in the median eminence of fasted mice: representative photomicrographs (D and E) and quantitative CRF-IR (green) cell number (A) and mean intensity (B and C) in both PVN and median eminence of WT or *Agrp-IRES-Cre* animals that received intra ARC injection of AAV-DIO-hM4D(Gi)-mCherry (red) submitted to 36 h of fasting and treated 5 i.p. injections CNO (1 mg/kg). Tissue samples obtained from experimental procedure described in the Fig. 3 were used for CRF immunostaining studies. The cell nuclei were staining with Hoechst (blue). Data are expressed as mean ± SEM and were analyzed Unpaired t test: ^#^p < 0.05 *vs* WT*::*AAV-DIO- hM4D(Gi)-mCherry fasted and treated with CNO.

## Data Availability

All the data related to this manuscript are available with the corresponding author.
