# Correction to: Arcuate AgRP, but not POMC neurons, modulate paraventricular CRF synthesis and release in response to fasting

**DOI:** 10.1186/s13578-022-00885-5

**Published:** 2022-09-03

**Authors:** Alan Carlos Alves Fernandes, Franciane Pereira de Oliveira, Gimena Fernandez, Luane da Guia Vieira, Cristiane Gugelmin Rosa, Taís do Nascimento, Suzelei de Castro França, Jose Donato, Kristen R. Vella, Jose Antunes-Rodrigues, André Souza Mecawi, Mario Perello, Lucila Leico Kagohara Elias, Rodrigo Rorato

**Affiliations:** 1 Department of Biotechnology, University of Ribeirao Preto, Ribeirão Prêto, SP 14096-900 Brazil; 2 Department of Physiology, Ribeirao Preto Medical School, University of Sao Paulo, Ribeirão Prêto, SP 14049-900 Brazil; 3 Department of Physiology and Biophysics, Institute of Biomedical Sciences, University of Sao Paulo, São Paulo, SP 05508-000 Brazil; 4 Department of Endocrinology, Diabetes and Metabolism and the Weill Center for Metabolic Health, Weill Cornell Medical College, New York, NY 10021 USA; 5 Laboratory of Neurophysiology of the Multidisciplinary Institute of Cell Biology [IMBICE, Argentine Research Council (CONICET) and Scientific Research Commission, Province of Buenos Aires (CIC-PBA), National University of La Plata, 403 La Plata, Buenos Aires Argentina; 6 Department of Biophysics, Paulista Medical School, Federal University of Sao Paulo, São Paulo, SP CEP 04023-062 Brazil

## Correction to: Cell & Bioscience (2022) 12: 118 https://doi.org/10.1186/s13578-022-00853-z

In the original version of this article, the given and family name of the co-author André Mecawi (Mecawi A) was incorrectly structured. The correct name is André Souza Mecawi (Mecawi AS). This has been corrected with this correction.

The original article [1] has been corrected.
