# Supplementary figures and images for: Arcuate AgRP, but not POMC neurons, modulate paraventricular CRF synthesis and release in response to fasting
Source: Cell Biosci. 2022 Jul 28;12:118. doi: 10.1186/s13578-022-00853-z (PMC9331576; doi:10.1186/s13578-022-00853-z)

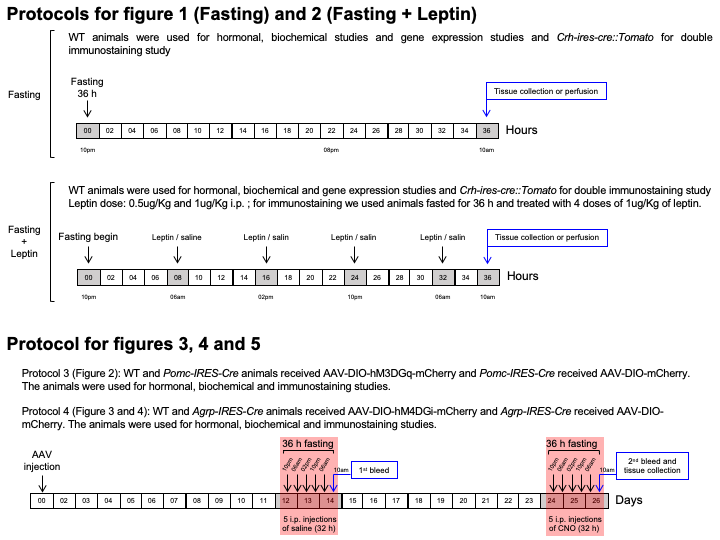

Supplement: Supplementary file 1 — Additional file 1: Figure S1. Experimental designs used in the protocols performed to obtain data showed in Fig. 1 (fasting and fasting + leptin studies) and Figs. 2, 3 and 4. [file 13578_2022_853_MOESM1_ESM.tiff]

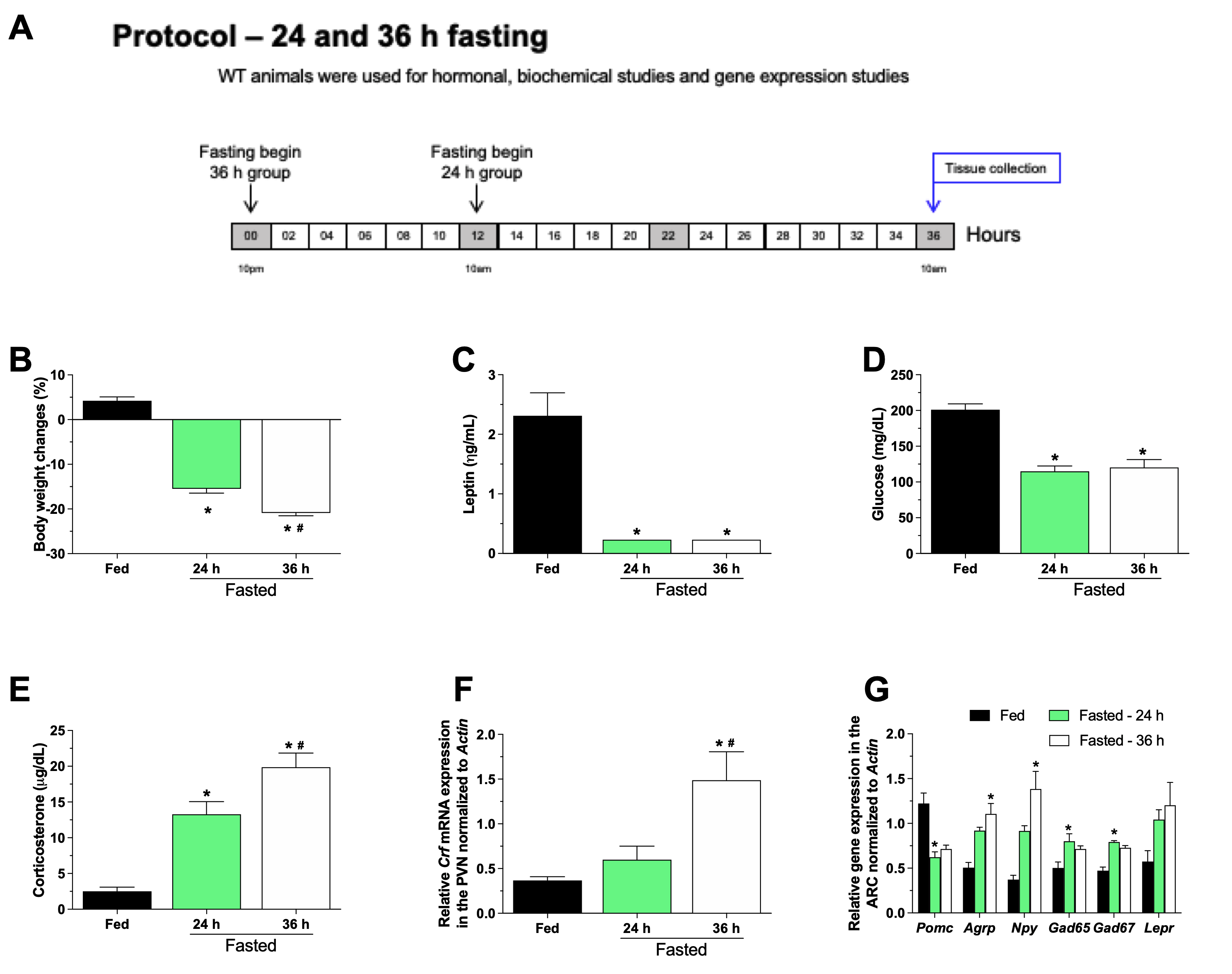

Supplement: Supplementary file 2 — Additional file 2: Figure S2. Prolonged fasting increases Crf mRNA expression in the PVN and induces higher corticosterone secretion: Experimental design (A), body weight change (B), plasma leptin (C), glucose (D) and corticosterone (E) levels, changes in the mRNA expression in the PVN (F) and ARC (G) of adult (8–10 weeks) WT mice fed or fasted for 24 h or 36 h (n = 8–10). Data are expressed as mean ± SEM and were analyzed by one-way ANOVA and Tukey test for multiple comparisons were used for samples that passed in the normality test (B, C, D and E) otherwise Kruskal–Wallis test followed by Dunn’s multiple comparisons were used (F and G): *p < 0.05 vs fed and #p < 0.05 vs 24 h fasting. [file 13578_2022_853_MOESM2_ESM.tiff]

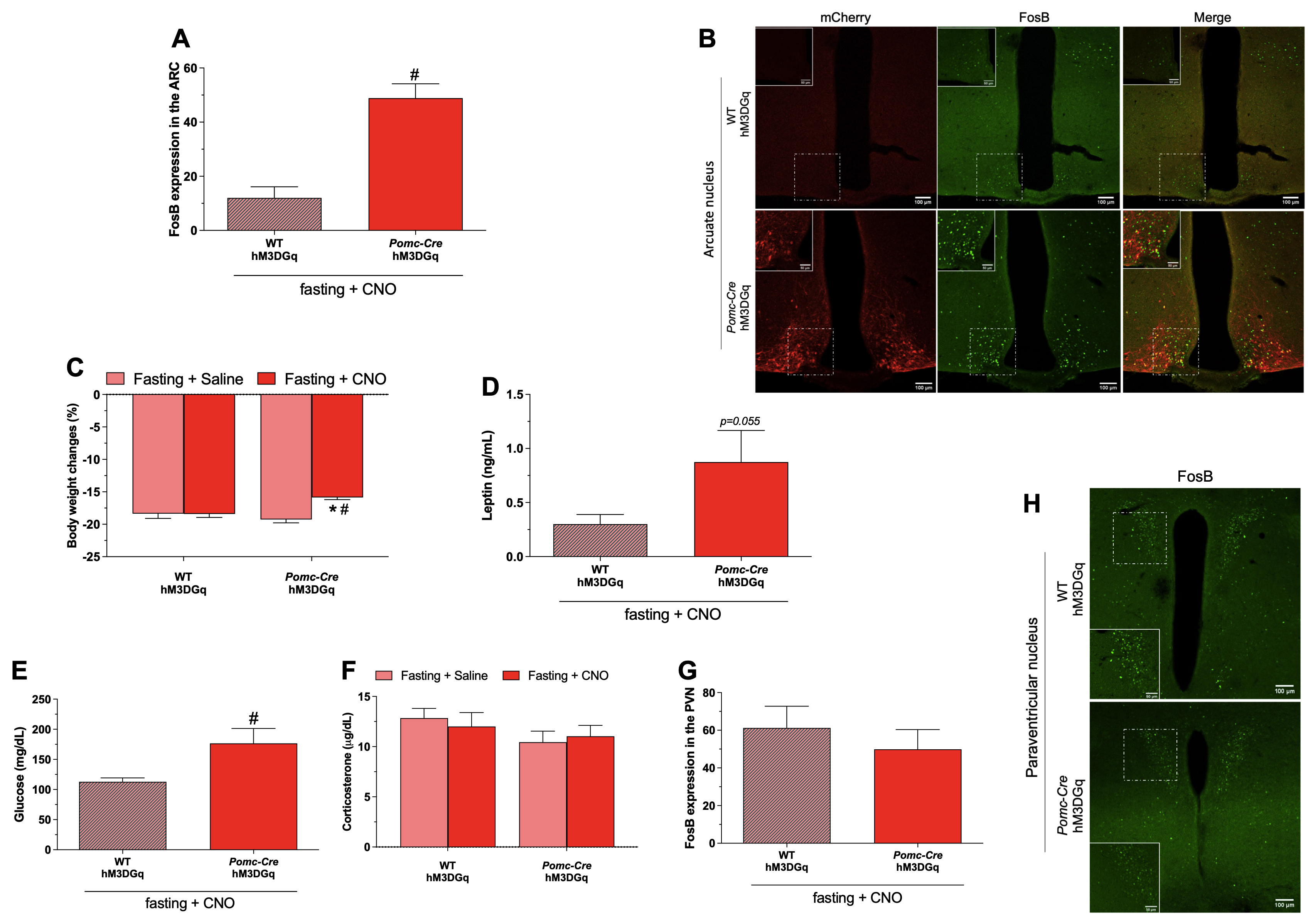

Supplement: Supplementary file 3 — Additional file 3: Figure S3. Chemogenetic activation of POMCARC neurons do not modulate fasting-induced HPA axis activation: FosB expression in the ARC (A), representative photomicrographs (10× and insert 20×) of double immunostaining mCherry/FosB in the ARC (C), body weight change (D), plasma leptin (E), glucose (F) and corticosterone (G) levels, PVN FosB expressing neurons (H) and representative photomicrographs (10× and insert 40×) of FosB in the PVN (I) of adult (8–12 weeks) male WT or Pomc-Cre mice (n = 8–18) that received intra ARC injection AAV-DIO-hM3D(Gq)-mCherry submitted to 36 h of fasting and treated with 5 i.p. injections CNO (1 mg/kg) or saline. The i.p. injections were performed each 8 h and the animals were euthanized at 10am as described in the Additional file 1: Fig. S1. Data are expressed as mean ± SEM and were analyzed by two-way ANOVA followed by post hoc Tukey’s multiple comparison (C and F) or unpaired t test. *p < 0.05 vs saline; #p < 0.05 vs Pomc-Cre::AAV-DIO-mCherry. [file 13578_2022_853_MOESM3_ESM.tiff]

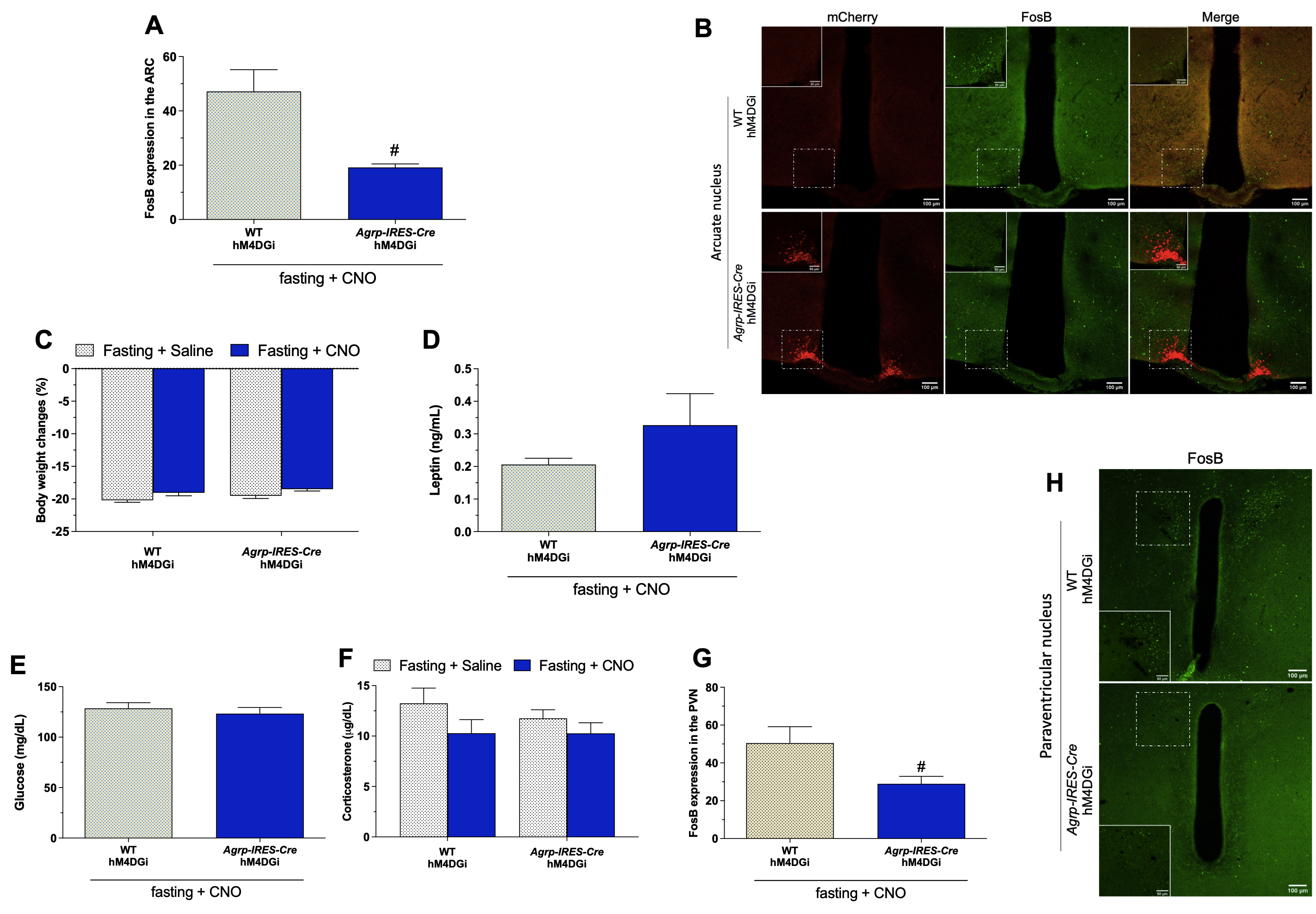

Supplement: Supplementary file 4 — Additional file 4: Figure S4. Chemogenetic inhibition of AgRPARC neurons reduces PVN neuronal activity without affecting plasma corticosterone levels in fasted animals: FosB expression in the ARC (A), representative photomicrographs (10× and insert 20×) of double immunostaining mCherry/FosB in the ARC (C), body weight change (D), plasma leptin (E), glucose (F) and corticosterone (G) levels, PVN FosB expressing neurons (H) and representative photomicrographs (10× and insert 40×) of FosB in the PVN (I) of adult (8–12 weeks) male WT or Agrp-IRES-Cre mice (n = 8–18) that received intra ARC injection AAV-DIO-hM4D(Gi)-mCherry submitted to 36 h of fasting and treated with 5 i.p. injections CNO (1 mg/kg) or saline. The i.p. injections were performed each 8 h and the animals were euthanized at 10am as described in the Additional file 1: Fig. S1. Data are expressed as mean ± SEM and were analyzed by two-way ANOVA followed by post hoc Tukey’s multiple comparison (C and F) or unpaired t test. *p < 0.05 vs saline; #p < 0.05 vs Agrp-IRES-Cre::AAV-DIO-mCherry. [file 13578_2022_853_MOESM4_ESM.tiff]

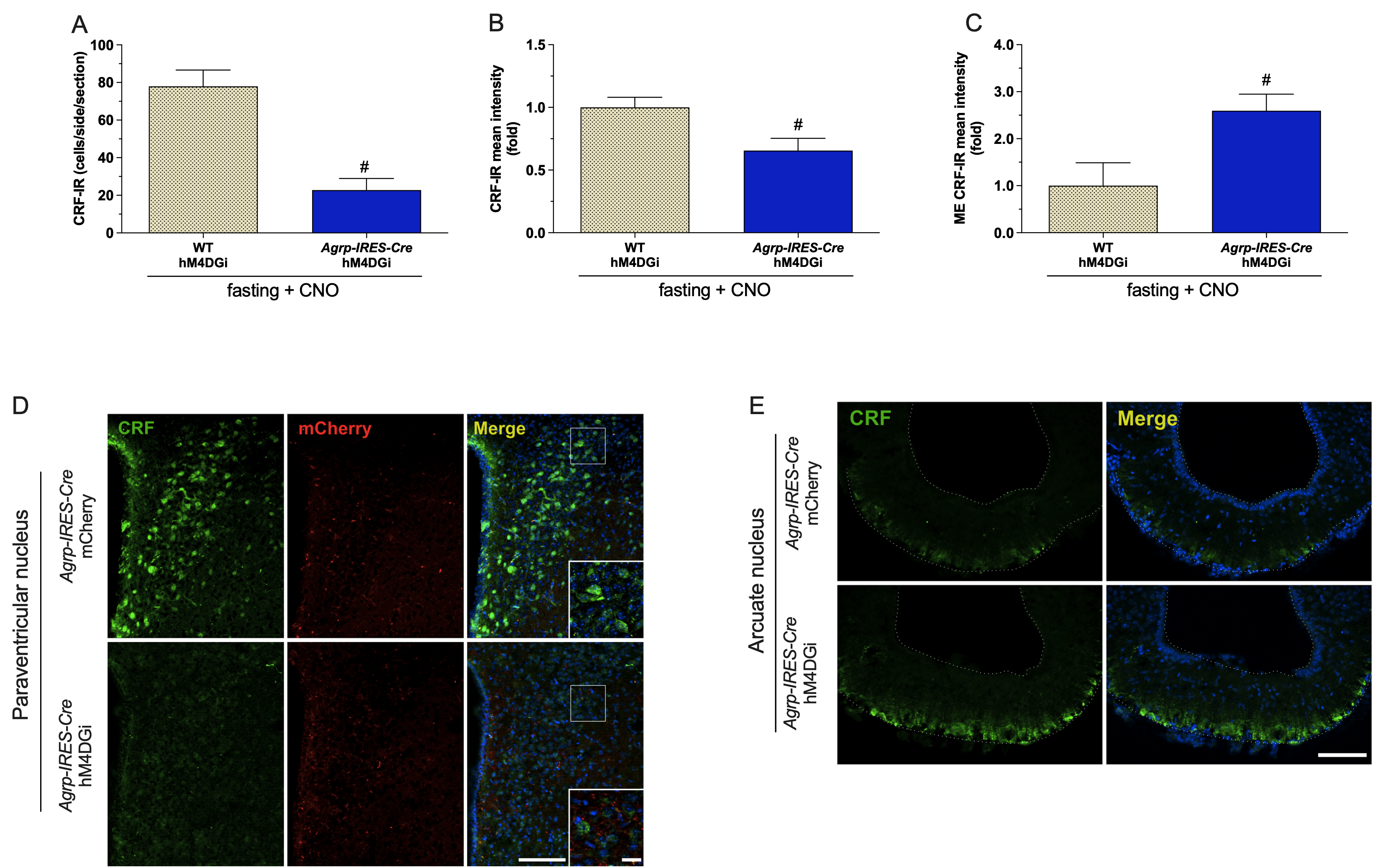

Supplement: Supplementary file 5 — Additional file 5: Figure S5. Inhibition of AgRPARC neurons reduces PVN CRF content and induces its accumulation in the median eminence of fasted mice: representative photomicrographs (D and E) and quantitative CRF-IR (green) cell number (A) and mean intensity (B and C) in both PVN and median eminence of WT or Agrp-IRES-Cre animals that received intra ARC injection of AAV-DIO-hM4D(Gi)-mCherry (red) submitted to 36 h of fasting and treated 5 i.p. injections CNO (1 mg/kg). Tissue samples obtained from experimental procedure described in the Fig. 3 were used for CRF immunostaining studies. The cell nuclei were staining with Hoechst (blue). Data are expressed as mean ± SEM and were analyzed Unpaired t test: #p < 0.05 vs WT::AAV-DIO- hM4D(Gi)-mCherry fasted and treated with CNO. [file 13578_2022_853_MOESM5_ESM.tiff]
